# The Effect of Baru (*Dypterix alata* Vog.) Almond Oil on Markers of Bowel Habits in Hemodialysis Patients

**DOI:** 10.1155/2021/3187305

**Published:** 2021-05-26

**Authors:** Raquel M. Schincaglia, Gustavo D. Pimentel, Maria do Rosário G. Peixoto, Lilian Cuppari, João Felipe Mota

**Affiliations:** ^1^Clinical and Sports Nutrition Research Laboratory (Labince), Faculty of Nutrition, Federal University of Goiás (UFG), Goiânia 740303-060, Brazil; ^2^Faculty of Nutrition, Federal University of Goiás (UFG), Goiânia 740303-060, Brazil; ^3^Division of Nephrology, Federal University of São Paulo and Oswaldo Ramos Foundation, São Paulo 04021-001, Brazil

## Abstract

Gastrointestinal symptoms are common in patients in hemodialysis treatment and were frequently associated with low intake of dietary fibers and liquids, oral iron supplementation, phosphate binders, and low level of physical activity. Thus, the aim of this study was to evaluate the effect of baru almond oil in comparison with mineral oil supplementation on bowel habits of hemodialysis patients. Thirty-five patients on hemodialysis (57% men, 49.9 ± 12.4 years) were enrolled in a 12-week single-blind clinical trial. Patients were allocated (1 : 2) by sex and age into (1) the mineral group: 10 capsules per day of mineral oil (500 mg each) or (2) the baru almond oil group: 10 capsules per day of baru almond oil (500 mg each). Bowel habits were assessed by the Rome IV criteria, Bristol scale, and self-perception of constipation. Food consumption, physical activity level, and time spent sitting were also evaluated at the baseline and at the end of the study. After 12 weeks of supplementation, the baru almond oil group showed reduced Rome IV score (6.1 ± 5.5 vs 2.8 ± 4.3, *p*=0.04) and the straining on the evacuation score (1.2 ± 1.4 vs 0.4 ± 0.7; *p*=0.04), while the mineral group did not show any change in the parameters. The frequency of self-perception of constipation was lower in the baru almond oil group after intervention (45.0% vs 15.0%, *p*=0.04). Baru almond oil improved bowel habit and the straining on evacuation in hemodialysis patients.

## 1. Introduction

Gastrointestinal symptoms are common in patients with chronic kidney disease (CKD) [1]. According to Carrera-Jiménez et al. [2], 90% of dialysis patients reported, at least, one gastrointestinal symptom. Bowel habit alterations are generally associated with a low intake of dietary fibers and liquids, oral iron supplementation, phosphate binders, and low a level of physical activity; however, such conduct and behaviors are frequently part of hemodialysis (HD) patients' treatment [3]. The chronic use of medicines to improve bowel habits is also considered a trigger for its alterations, since they can alter bowel mucous secretions and motility [3]. In addition, peristalsis-stimulating medicines and osmotic drugs are contraindicated because they are a source of phosphates, sodium, and magnesium, which may compromise the HD treatment [4].

Thus, alternative approaches to improve bowel habits are required. The mineral oil is considered the main strategy because it is a feces emollient [5–8]. However, it has been observed that dietary oils offered in a liquid form, such as olive oil and flaxseed oil, have similar effects to mineral oil [4, 9] or even better effects by increasing the frequency of evacuation in constipated patients who consumed olive oil [4]. The probable mechanism of action of almond oils is softening feces, improving consistency, stimulating peristalsis, and reducing intestinal transit time [10].

Another advantage of using seeds or nuts oils is the fatty acids pattern [4], which may also improve other aspects of the health of patients with CKD, such as oxidative stress, inflammation, and lipid profile [11–16]. From this perspective, the baru almond (Dipteryx alata Vog.) has been gaining prominence [17, 18]. This almond has important lipid content [18], and in a recent study conducted by our group, baru almonds improved lipid profile and adiposity in overweight women [19]. However, no study with CKD or addressing bowel habits with this almond has been performed. Thus, the aim of this study was to evaluate the effect of baru almond oil in comparison with mineral oil supplementation on bowel habits of patients undergoing hemodialysis.

## 2. Materials and Methods

### 2.1. Patients

Patients with CKD on HD were recruited from two dialysis centers into a single-blind, controlled clinical trial for 12 weeks. The inclusion criteria were age between 25–69 years, length on HD greater than 3 months, dialysis access by arteriovenous fistula, body mass index (BMI) greater than 20 kg/m^2^, and Kt/V > 1.2. The exclusion criteria were history of gastrointestinal inflammatory disease, chronic use of laxatives, recent hospitalization (<3 months), poorly controlled diabetes, thyroid dysfunction, and intolerance to baru almond or allergy to any nut. This research and amendment project were approved by the Research Ethics Committee of the Federal University of Goias under the protocol 1.007.104/2015 and 2.520.652/2018 and registered in the Brazilian Registry of Clinical Trials (REBEC) under number RBR-3hj4ny. All participants were informed about the study and gave written informed consent to participate.

### 2.2. Study Protocol

Participants were randomly assigned (2 : 1; stratified by sex and age) to receive 10 capsules daily containing 500 mg of baru almond oil (BG) or 500 mg mineral oil (MG). Patients were instructed to consume five capsules after lunch and dinner. Capsules were used to ensure the intake of the recommended amount of oil and the blind design. Capsule intake follow-up was performed weekly asking directly to the patient during the HD sessions. The total capsule consumption greater than 75% was considered adequate for inclusion of the patient in the statistical analysis.

The cold-extracted baru oil was entirely composed of fatty acids, mostly of monounsaturated fatty acids (46.2%) followed by saturated (27.0%) and polyunsaturated fatty acids (26.8%). The fatty acids present in the baru almond oil were dosed from their esterification according to the method used by Shirai et al. [20], using gas chromatography coupled to the mass spectrometer GCMS-QP2010-Ultra, Shimadzu®). The mineral oil was composed entirely of liquid paraffin. Both oils were encapsulated in gelatinous capsules.

#### 2.2.1. Bowel Habit

Bowel habit was assessed by the Rome IV criteria before and after the intervention. The six symptoms evaluated were less than three bowel movements/week, straining on evacuation, presence of lumpy or hard stools, sensation of incomplete evacuation, anorectal obstruction, and the use of manual maneuvers to facilitate evacuation. These symptoms are scored according to their frequency as 0 = never or rarely, 1 = sometimes, 2 = often, 3 = most of the time, and 4 = always. For diagnostic purposes, the frequency of symptoms must be present, at least, sometimes or often. The sum of scores composes the general score for the Rome IV score, ranging from 0 to 24 points.

The patients were diagnosed as constipated when presented at least two criteria of Rome IV with *e* with the frequencies most of the time or always. Patients were also questioned about their subjective self-perception about constipation (self-reported constipation). A question was asked directly to the patient: “Do you consider yourself constipated?,” with two answer options: “Yes” or “No”.

#### 2.2.2. Stool Consistency

The stool consistency was self-evaluated using images from the Bristol scale on the week prior to the beginning of intervention and at the end of intervention. The participants should report the average stool consistency in the week prior to data collection. The seven categories (ordinal variable) of this scale are 1- “separated and hard pieces like peanuts,” 2- “form of sausage but segmented,” 3- “form of sausage but with slits in the surface,” 4- “form of sausage or smooth snake and soft” (adequate form), 5- “soft pieces with rigid contours,” 6- “aerated pieces and ripped contours,” and 7-“aqueous without solid pieces” [21, 22].

#### 2.2.3. Anthropometry and Laboratory Parameters

Anthropometry was performed using standardized procedures shortly after the intermediate HD session of the week [23]. Current body weight (kg) and height (*m*) were measured and used to calculate body mass index (BMI, kg/m^2^).

Serum urea (ultraviolet kinetic method), potassium (selective electrode method), total calcium (colorimetric method of end point), and phosphorus (photometric method) were performed with samples collected before the intermediate HD session of the week (AU5800 Series Chemistry Analyzers, Beckman Coulter, Pasadena, California, USA). The Kt/V was calculated according to Daugirdas II [24].

#### 2.2.4. Dietary Intake, Physical Activity, and Medications

The dietary intake was assessed by nonconsecutive three-day 24 hour recalls (one day of HD, one without HD, and one day of the weekend, specifically Sunday), at baseline and at the end of the intervention [23], according to the USDA's Automated Multiple-Pass Method [25, 26]. The 24 hour recalls were collected at the time of initial supplementation and in the last week of supplementation. Data were processed using AVANUTRI® software (Rio de Janeiro, Brazil), and the total calories, carbohydrate (grams and %), protein (grams, %, and grams/kg/day), lipid (grams and %), and fiber intake (grams and grams/1000 kcal/day) were estimated. The liquid balance was evaluated considering the volume of ingested liquids reported by the patient and subtracted from residual urine volume. Medications that contribute to constipation were evaluated from the medical prescription and patients' report.

Physical activity level was evaluated by the International Questionnaire of Physical Activity-short version [27]. Total energy expenditure was estimated by calculating the metabolic equivalent of a task (MET/minute/week) according to the results obtained in the questionnaire. The average sitting time was estimated with the evaluation of the patient's routine in three days, one day with HD, one day without HD, and one day on the weekend.

### 2.3. Statistical Analysis and Sample Size

A Shapiro–Wilk test was performed for testing normality. Variables are expressed as mean and standard deviation or frequency. The comparisons between characteristics of the groups at baseline were performed using unpaired Student's *t*-test. For the nonhomoscedastic variables between groups at the baseline (Rome IV score and Bristol scale score), an adjustment of the final data based on the baseline data was performed using a linear model of regression, making it an homoscedastic variable. The comparisons between the moments (pre- and postsupplementation) and between groups (mineral group and baru almond oil group) were performed by the Mann–Whitney test due to the absence of normality or the use of ordinal variables.

For the categorical variables, the Fisher Exact test was performed. Statistical analyzes were performed with STATA 14.0 software. The agreement between the evaluation of the Rome IV questionnaire and the self-reported constipation was performed by the Kappa test. The level of significance was set at 5% (*p* < 0.05).

The effect size was estimated by the Cohen *d* test from the calculation of differences between groups and between baseline and final moments. The power of the test (1-*β*) was estimated a posteriori equal to 0.83 (83%), considering the variable Rome IV score and the variations of its means of the final moment in relation to the baseline, Mann–Whitney test, two-tailed, and an alpha of 0.05.

## 3. Results

**Figure 1:**
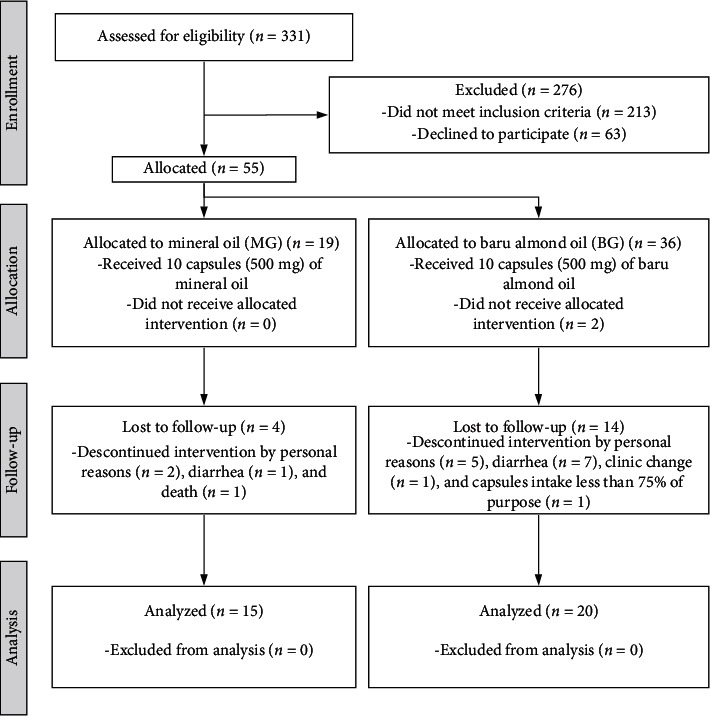
Participant flow through the study.

As shown in Figure 1, from 331 patients evaluated for eligibility, 276 patients did not meet the inclusion criteria or refused to participate, resulting in a sample of 55 individuals. Two patients did not receive the intervention because they declined for personal reasons, and during the follow-up, 18 patients were excluded due to diarrhea (*n*=8), personal reasons (*n*=7), death (*n*=1), clinic change (*n*=1), and poor adherence to the supplementation (<75%, *n*=1) Therefore, 35 patients completed the study, 15 patients in the MG and 20 in the BG.

The groups were homogeneous regarding demographic and clinical parameters (Table 1). The mean age was 49.9 ± 12.4 years, most of the patients were male (57.1%), and the BMI was 25.9 ± 4.5 kg/m^2^. The main comorbidities among the patients were hypertension (80.0%) and diabetes (28.6%).

At baseline, the symptoms reported by patients were lumpy or hard stools (MG 6.7% vs. BG 30.0%, *p*=0.32), straining on evacuating (MG 20.0% vs. BG 20.0%, *p* > 0.99), three bowel movements/week (MG 13.3% vs. BG 20.0%, *p* > 0.99), sensation of incomplete evacuation (MG 13.3% vs. BG 15.0%, *p* > 0.99), anorectal obstruction (MG 13.3% vs. BG 10.0%, *p* > 0.99), and manual maneuvers to facilitate evacuation (MG 0 vs. BG 10.0%, *p*=0.41). According to the Rome IV criteria, 37.1% of patients were diagnosed with constipation at baseline with no difference between the groups. The prevalence of constipation did not change after the intervention in both groups (MG 33.3% vs. 6.7%, *p*=0.17; BG 40.0% vs. 25.0, *p*=0.25). The concordance between the evaluation of the Rome IV questionnaire and the self-reported constipation estimated a kappa of 0.51 (*p* < 0.001) with an agreement percentage of 80% between the evaluations.

It was observed that 77.14% of the patients used medications and/or supplements which cause constipation, of which 65.00% was in the BG and 93.33% was in the MG, but no differences were found between groups (*p*=0.19). There were no changes in medication and supplement use in both groups during the study. The adherence rates were 84.7% in the BG and 84.9% in the MG, respectively (*p*=0.93). Diarrhea was the most frequent side effect reported during the intervention and was reported by 15.8% and 25.6% of patients from the MG and BG, respectively. Laboratory parameters did not differ within and between groups. Likewise, no changes were observed in food and liquid intake and physical activity level (*p* > 0.05). However, sitting time was higher in the BG compared to the MG (Table 2).

**Figure 2:**
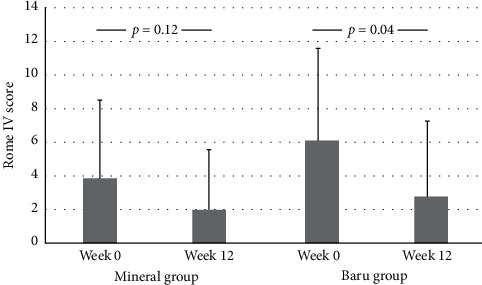
Roma IV score before (week 0) and after intervention (week 12) according to the groups, mineral group and baru almond oil group. Data are expressed as mean ± standard deviation. The *p* value was obtained by the Mann–Whitney *U*-test, with a 5% level of significance.

After 12 weeks of supplementation, the Rome IV score decreased only in the BG group (6.1 ± 5.5 vs 2.8 ± 4.3; *p*=0.04) (Figure 2). No difference was found in bowel habits in the MG, while the straining on the evacuation score was reduced in the BG (Table 2). The frequency of self-perception constipation reduces after baru oil supplementation (45.0% vs. 15.0%; *p*=0.04).

## 4. Discussion

In the present study, we showed, for the first time, the potential beneficial effect of baru almond oil to improve bowel habits in patients on hemodialysis. This effect was independent of dietary fiber intake and physical activity level. Alterations in bowel habits, especially constipation, are often reported by CKD patients under dialysis therapy [3, 28–30].

Healthy bowel habits are related to lifestyle changes [30–32]. One of them is the increase in fiber intake, which is generally low among patients with end-stage kidney disease. Low fiber intake is due to the fact that many fiber-rich foods are also high in phosphorus and/or potassium and micronutrients which should be restricted in their diets [3]. In the present study, fiber intake was higher than that in the study conducted by Yasuda et al. [29] (5.9 ± 2.7 g per day).

Another contributing factor for inadequate bowel habits is low liquid intake [30]. Water restriction is important to avoid retention of body fluids, control blood pressure, and prevent cardiovascular complications [23]. However, adequate liquid intake is important for peristalsis and intestinal lubrication [3].

Physical exercise is also important for bowel habit regulation, since it promotes acceleration of bowel peristaltic movements [30]. However, hemodialysis patients are less physically active due to exercise intolerance and occupational tasks, unemployment, sarcopenia, impaired functional capacity, and decreased quality of life [3, 28]. This aspect was also verified in our study, since the majority of the patients have a sedentary behavior, with low physical activity level and significant amounts of time spent sitting per day.

Mineral oil, made up of liquid paraffin, is frequently used, and several studies showed positive effects on bowel function [5–7, 31]. In addition, mineral oil may have an effect superior to treatment with lactulose [33]. As far as we know, only one study showed a favorable effect of mineral oil on constipation in patients on hemodialysis [4]. Indeed, mineral oil supplementation (mean of 5.7 g/day) promoted a reduction in the frequency of constipation and an improvement in the number of bowel movements, hard stools, the need for force to evacuate, the sensation of incomplete evacuation, and intestinal obstruction [4]. This is in contrast with the present study since we did not observe any change in bowel habits markers. This may be due to the fact that, in this study, we included patients regardless of their Rome IV score, while only constipated patients were investigated in the study performed by Ramos et al. [4].

Although the chronic use of mineral oil appears to be quite safe and has little or no adverse systemic effect [7], new treatment possibilities for improving bowel function have been sought with the perspective that new substances may have other potential beneficial effects. In this perspective, two studies with patients with CKD were found using natural oils, one with olive oil [9] and the other with olive oil and flaxseed oil [4]. The first study aimed to evaluate the effect of olive oil optimized by mixing different oils on biochemical parameters and nutritional status of predialytic patients in stage 4-5 of CKD. The patients received 60 mL daily of the modified oil for 30 days, and as a side effect, a reduction of constipation was observed (100%). Despite the dose, the authors reported good oil tolerance [9]. The second study was performed with constipated patients undergoing HD, and the authors observed that olive (on average 5.7 mL) or flaxseed oils (on average 6.9 mL) were efficient in reducing aspects of constipation. However, only olive oil was associated with an improvement in the need for force when evacuating, a sensation of incomplete evacuation, and in anorectal obstruction, suggesting that olive oil was superior to flaxseed and mineral oils for the treatment of constipation [4]. Another positive effect with the use of natural oils is that they can provide calories and help in the treatment of patients with low body weight.

In our study, baru almond oil promoted a reduction in alterations of the bowel habits (assessed by the Rome IV score) and the force required for evacuating in hemodialysis patients. On the other hand, mineral oil did not modify any aspect of bowel habits. In addition, it is worth mentioning that natural oils may have other superior effects to mineral oil because they are a source of monounsaturated and polyunsaturated fatty acids known to be related to in the improvement of lipid profile, inflammation, oxidative stress, and body composition [11–15, 18, 19, 34]. Unfortunately, in the present study, we were not able to measure these outcomes.

It is noteworthy that, in our study, none of the groups modified the fiber intake and physical activity level, or changed their drug therapy, demonstrating the real effect on bowel habits during the intervention. We further verified that there were no changes in Kt/V, urea, potassium, phosphorus, and serum calcium, suggesting that supplementation with 5g of the baru almond and mineral oils was safe for chronic renal patients in HD.

Some limitations of the present study were the evaluation of liquid intake only by 24vhour recalls and the exclusion of patients in chronic use of laxative medications what might have contributed to a larger number of constipated patients raising the possibility to obtain more robust results about the potential beneficial effect of baru oil on bowel alterations.

In addition, it is suggested that larger doses could have a greater effect on bowel habits, opening the possibility of further studies in which a maximum dose or an ideal dose is tested to modify all aspects of bowel habits in chronic renal patients of HD.

## 5. Conclusions

After 12 weeks of supplementation, the baru almond oil group showed reduction in the Rome IV score and the straining on evacuation, while the mineral group did not show any change in the parameters. The frequency of self-perception of constipation was lower in the baru almond oil group after intervention. Our study uses baru almond oil as a potential alternative treatment for alterations in bowel habits in HD patients and is among the first studies looking for the use of natural oils for evaluation in the intestinal health of these patients. We conclude that daily consumption of 5g of baru almond oil was safe and efficient to improve bowel habits and the score of straining to evacuating in hemodialysis patients.

## Figures and Tables

**Table 1:** Characterization at baseline.

	Total sample *n* = 35	Mineral oil group *n* = 15	Baru almond oil group *n* = 20	*p* value
Age	49.9 ± 12.4	51.7 ± 11.8	48.6 ± 12.9	0.47^*T*^
Adult	27 (77.1)	12 (80.0)	15 (75.0)	1.00^*∗*^
Elderly	8 (22.9)	3 (20.0)	5 (25.0)	

Sex				
Male	20 (57.1)	10 (66.7)	10 (50.0)	0.49^*∗*^
Female	15 (42.9)	5 (33.3)	10 (50.0)	

Current smoking habit				
Yes	2 (5.7)	1 (6.7)	1 (5.0)	1.00^*∗*^
No	33 (94.3)	16 (93.3)	19 (95.0)	

Current alcoholic habit				
Yes	7 (20.0)	3 (20.0)	4 (20.0)	0.71^*∗*^
No	28 (80.0)	12 (80.0)	16 (80.0)	

Body mass index (kg/m^2^)	25.9 ± 4.5	26.4 ± 5.5	25.5 ± 3.7	0.96^*T*^

Comorbidity				
Diabetes	10 (28.6)	5 (33.3)	5 (25.0)	0.71^*∗*^
Hypertension	28 (80.0)	11 (73.3)	17 (85.0)	0.43^*∗*^

Etiology of chronic kidney disease				
Diabetes	8 (22.9)	3 (20.0)	5 (25.0)	1.00^*∗*^
Hypertension	16 (45.7)	6 (40.0)	10 (50.0)	0.73^*∗*^

Values are presented in mean ± standard deviation or absolutes (*n*) and relatives (%) frequencies. The *p* value was obtained by the ^*∗*^Exact Fisher test or, ^*T*^t-Student unpaired test, 5% level of significance.

**Table 2:** Bowel habit, laboratory parameters, food consumption, and physical activity variables at the baseline and final moments of the study for the mineral oil and baru almond oil groups.

	Mineral group (*n* = 15)			Baru almond oil group (*n* = 20)			*p* value, baseline	*p* value, final	Effect size
	Week 0	Week 12	*p* value	Week 0	Week 12	*p* value			
*Bowel habits*									
<3 bowel movements/week^‡^	0.7 ± 1.4	0.5 ± 1.4	0.44	1.2 ± 1.6	0.6 ± 1.2	0.16	0.38	0.68	1.67
Hard or lumpy stools^‡^	0.5 ± 0.8	0.5 ± 1.1	0.50	1.4 ± 1.5	0.8 ± 1.2	0.10	0.06	0.39	0.69
Straining on evacuation^‡^	0.9 ± 1.3	0.3 ± 0.6	0.33	1.2 ± 1.4	0.4 ± 0.7	**0.04**	0.31	0.59	1.57
Incomplete evacuation^‡^	0.9 ± 1.0	0.5 ± 0.8	0.17	1.2 ± 1.4	0.6 ± 1.0	0.09	0.60	0.84	0.36
Anorectal obstruction^‡^	0.6 ± 1.0	0.1 ± 0.5	0.08	0.7 ± 1.1	0.3 ± 0.6	0.18	0.58	0.19	0.27
Manual maneuvers^‡^	0.3 ± 0.6	0 ± 0	0.07	0.4 ± 0.7	0.1 ± 0.4	0.40	0.75	0.12	0.29
Bristol Scale score^‡^	4.1 ± 1.0	4.4 ± 1.2^*∗∗*^	0.95	3.5 ± 1.5	4.2 ± 0.6^*∗∗*^	0.21	**0.05**	0.70	4.50
Constipation self-perception, *n*(%)^*∗*^	4 (26.7)	5 (33.3)	1.00	9 (45.0)	3 (15.0)	**0.04**	0.31	0.25	---

*Laboratory parameters*									
Kt/V^†^	1.5 ± 0.4	1.5 ± 0.3	0.71	1.6 ± 0.3	1.6 ± 0.3	0.66	0.65	0.59	0.04
Urea (mg/dL)^†^	114.6 ± 27.0	129.0 ± 39.0	0.25	124.3 ± 25.6	123.8 ± 28.9	0.89	0.28	0.66	0.82
Potassium (mg/dL)^‡^	5.8 ± 2.9	5.1 ± 0.4	0.88	6.0 ± 3.1	5.4 ± 0.6	0.83	0.43	0.13	0.65
Phosphorus (mg/dL)^†^	4.6 ± 1.2	4.9 ± 1.4	0.60	4.8 ± 1.4	5.1 ± 1.2	0.64	0.56	0.76	0.02
Calcium (mg/dL)^†^	9.5 ± 0.6	9.1 ± 0.6	0.07	9.1 ± 0.8	9.0 ± 0.8	0.65	0.25	0.87	1.57

*Food consumption and physical activity level*									
Calories (Kcal)	1267.0 ± 531.7	1216.1 ± 370.4	0,85	1372.6 ± 591.2	1331.9 ± 533.1	0.96	0.57	0.57	0.25
Carbohydrate (g)	144.9 ± 53.3	154.7 ± 54.2	0.57	168.1 ± 73.4	160.2 ± 71.7	0.79	0.46	0.95	0.09
Carbohydrate (%)	49.6 ± 9.4	51.3 ± 8.8	0.57	50.61 ± 5.9	47.7 ± 6.6	0.21	0.84	0.20	0.46
Protein (g)	69.8 ± 48.1	55.7 ± 20.1	0.72	60.6 ± 25.0	56.9 ± 25.1	0.61	0.95	0.97	0.05
Protein (%)	18.6 ± 3.7	18.2 ± 2.9	0.73	17.7 ± 4.4	17.3 ± 3.7	0.82	0.32	0.76	0.27
Protein (g/kcal/day)	1.0 ± 0.7	0.8 ± 0.3	0.78	0.9 ± 0.4	0.9 ± 0.4	0.96	0.88	0.74	0.28
Lipid (g)	55.8 ± 45.9	41.4 ± 16.1	0.72	50.9 ± 26.5	51.5 ± 20.7	0.64	0.84	0.15	0.54
Lipid (%)	31.7 ± 6.7	30.7 ± 6.3	0.65	31.7 ± 6.6	34.9 ± 5.3	0.09	0.91	0.06	0,72
Fiber intake (*g*)^‡^	11.5 ± 7.2	12.5 ± 6.3	0.47	12.6 ± 6.4	11.3 ± 6.7	0.42	0.44	0.51	0.47
Fiber intake (g/1000 Kcal/day)	9.2 ± 4.3	10.4 ± 3.8	0.27	9.5 ± 4.2	8.6 ± 3.9	0.40	0.64	0.16	0.47
Residual liquid intake (l)	0.7 ± 0.5	0.9 ± 0.5	0.28	0.7 ± 0.4	0.8 ± 0.5	0.50	0.66	0.35	1.0
Physical activity level (MET/min/week)^‡^	231.4 ± 268.2	296.6 ± 422.0	0.98	758.6 ± 765.6	1086.6 ± 1308.1	0.49	0.44	0.12	1.01
Sitting time (min/day)^‡^	521.1 ± 162.4	484.8 ± 125.1	0.43	558.3 ± 174.8	606.3 ± 163.1	0.45	0.50	**0.03**	0.37

Values presented as mean ± standard deviation or absolute (*n*) and relative (%), and *p* values are obtained by ^*∗*^Fisher's exact test or ^‡^the Mann–Whitney *U*-test with 5% level of significance. ^*∗∗*^Data adjusted for the initial moment due to the absence of homoscedasticity. Effect size estimated by Cohen *d*.

## Data Availability

The data used to support the findings of this study are available from the corresponding author upon request.
